# A study on the differences in the gut microbiota and metabolism between male and female mice in different stress periods

**DOI:** 10.3389/ebm.2025.10204

**Published:** 2025-02-11

**Authors:** Yajun Qiao, Juan Guo, Qi Xiao, Jianv Wang, Xingfang Zhang, Xinxin Liang, Lixin Wei, Hongtao Bi, Tingting Gao

**Affiliations:** ^1^ Qinghai Provincial Key Laboratory of Tibetan Medicine Pharmacology and Safety Evaluation, Northwest Institute of Plateau Biology, Chinese Academy of Science, Xining, China; ^2^ School of Psychology, Chengdu Medical College, Chengdu, China; ^3^ CAS Key Laboratory of Tibetan Medicine Research, Northwest Institute of Plateau Biology, Chinese Academy of Sciences, Xining, China; ^4^ Qinghai Provincial Traffic Hospital, Xining, China; ^5^ Emergency Department, The First Hospital of Qinhuangdao, Qinhuangdao, China; ^6^ Medical College, Qinghai University, Xining, China; ^7^ Department of Psychiatry, The People’s Hospital of Jiangmen, Southern Medical University, Jiangmen, China

**Keywords:** stress, sex differences, gut microbiota, gut metabolism, estrogen metabolism

## Abstract

The sex difference in depression has long been an unsolved issue. Women are twice as likely to suffer from depression as men. However, there were significant differences in the composition of gut microbiota between women and men. There is a lack of studies linking sex differences in depression to microbiota, and the specific mechanisms of this process have not been explained in detail. The main purpose of this study was to explore the gender differences in the intestinal tract of male and female depressed mice. In this study, chronic restraint stress (CRS) mouse models were used to simulate chronic stress, and behavioral tests were conducted, including the open field test (OFT), tail suspension test (TST) and forced swimming test (FST). Microbial diversity analysis and metabolomics were performed on collected mouse feces. The results showed that female mice were highly active and prone to anxious behavior before stress, and the levels of *f-Rikenellaceae, f-Ruminococcaceae* and 16α-hydroxyestrone were significantly different from those in male mice. After 21 days (Days) of stress, female mice showed depression-like behavior, and the levels of *f-Erysipelotrichaceae*, 5α-pregnane-3,20-dione, and 2-hydroxyestradiol were significantly different from those in male mice. After 14 days of stress withdrawal, the depression-like behavior continued to worsen in female mice, and the levels of 5α-pregnane-3,20-dione, estrone glucuronide and *f-Erysipelotrichaceae* were significantly different from those in male mice. In summary, female mice have stronger stress sensitivity and weaker resilience than male mice, which may be related to differences in bacterial diversity and estrogen metabolism disorders.

## Graphical Abstract

**Figure:**
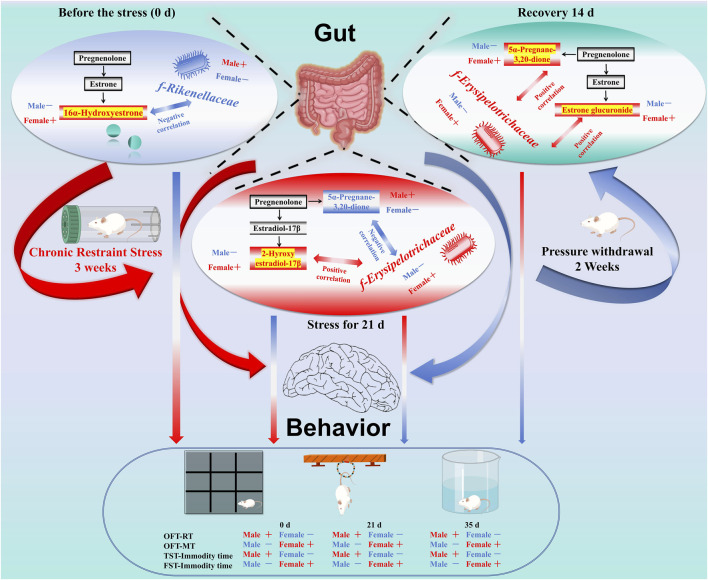
Note: Sex differences in depression have long been noted, with women being twice as likely to experience it compared to men. This study used chronic restraint stress (CRS) mouse models to investigate these differences, focusing on behavioral and microbial changes. Behavioral tests included the open field test (OFT), tail suspension test (TST), and forced swimming test (FST). Microbial diversity and metabolomics were analyzed from fecal samples. Results showed that female mice exhibited higher pre-stress activity and anxiety levels. Before stress, *f-Rikenellaceae*, *f-Ruminococcaceae*, and 16α-hydroxyestrone levels differed significantly between sexes. After 21 days of stress, female mice displayed depression-like behavior, with significant differences in *f-Erysipelotrichaceae*, 5α-pregnane-3,20-dione, and 2-hydroxyestradiol levels. Post-stress withdrawal for 14 days further exacerbated depression-like behavior in females, with continued differences in 5α-pregnane-3,20-dione, estrone glucuronide, and *f-Erysipelotrichaceae* levels. In summary, female mice show greater stress sensitivity and weaker resilience, potentially linked to bacterial diversity and estrogen metabolism differences.

## Impact statement

In the present study, our experiments confirmed that prior to stress, females showed higher activity, despair and fight capacity, anxiety-like behaviour, gut flora (*f-Rikenellaceae* and *f-Ruminococcaceae*) and 16α-hydroxyestrone, significantly different from males; after 21 days of stress, females had worsened anxiety and depression-like behaviours, and gut metabolism and microbiota were disrupted, with significant differences in microbiota (*f-Erysipelotrichaceae*) and metabolites (5α-pregnane-3, 20-dione and 2-hydroxyestradiol); and after 14 days of stress relief, males began to recover from depression-like behaviours, whereas females showed deterioration. These results may provide new insights into gender-specific treatment and prevention of depression in patients with depression.

## Introduction

Sex differences have consistently been a compelling area of scientific investigation. Notably, the divergence between males and females is particularly pronounced in both the prevalence and expression of depression. According to a 2021 study published in The Lancet, approximately 1 billion people worldwide are suffering from mental disorders [1]. The global burden of mental disorders has become heavier since the COVID-19 (coronavirus disease 2019) pandemic, with cases of major depression and anxiety increasing by 27.6% and 25.6%, respectively. The pressure of the epidemic has brought greater challenges to the diagnosis and treatment of depression [2]. Previous studies have shown that stress has the worst impact on women, with depression approximately twice as common in women as in men [3]. Currently, brain-gut axis communication is being studied as an important way to understand mental diseases, and gut microbes play an important role in brain and behavioral disorders [4–6].

Interestingly, there are also sex differences in the gut microbiota. B Flak, when commenting on the article published by JG Markle in Science in 2013, proposed the idea of sexual dimorphic microbiota and believed that there are differences in the microbiota of men and women [7, 8]. Li et al. found that 3β-hydroxysteroid dehydrogenase expressed by the gut microbiome can “eat” estradiol and is associated with depression in premenopausal women [9]. Estrogen also has physiological functions that interfere with pain regulation [10]. Existing studies have demonstrated a complex interaction between sex hormone signaling and stress responses in brain-gut axis function [11]. However, the specific mechanism of this process has not been elucidated in detail. Therefore, we used a CRS model to simulate stress in female and male mice and investigated the different behaviors of female and male mice in the face of stress through the differences in the gut microbiota and metabolites.

## Materials and methods

### Animals

Kunming (KM) mice (8 weeks of age) were obtained from Gansu University of Chinese Medicine, China [animal production license number: SCXK (Gan) 2015-0005]. Mice were housed in standard cages with wood shavings in a room with a carefully controlled ambient temperature (22 ± 1°C) and artificial lighting from 7:00 a.m. to 7:00 p.m. and were fed standard laboratory chow and provided distilled water *ad libitum*. All animal experiments were in compliance with the ARRIVE (Animal Research: Reporting of *In Vivo* Experiments) guidelines and were carried out in strict accordance with the National Institutes of Health Guide for the Care and Use of Laboratory Animals (NIH Publication No. 8023, revised 1978) and approved by the committee of the Northwest Plateau Institute of Biology, CAS, for animal experiments (allowance number: NWIPB20171106-01).

### Stress stimuli and animal grouping

**FIGURE 1:**
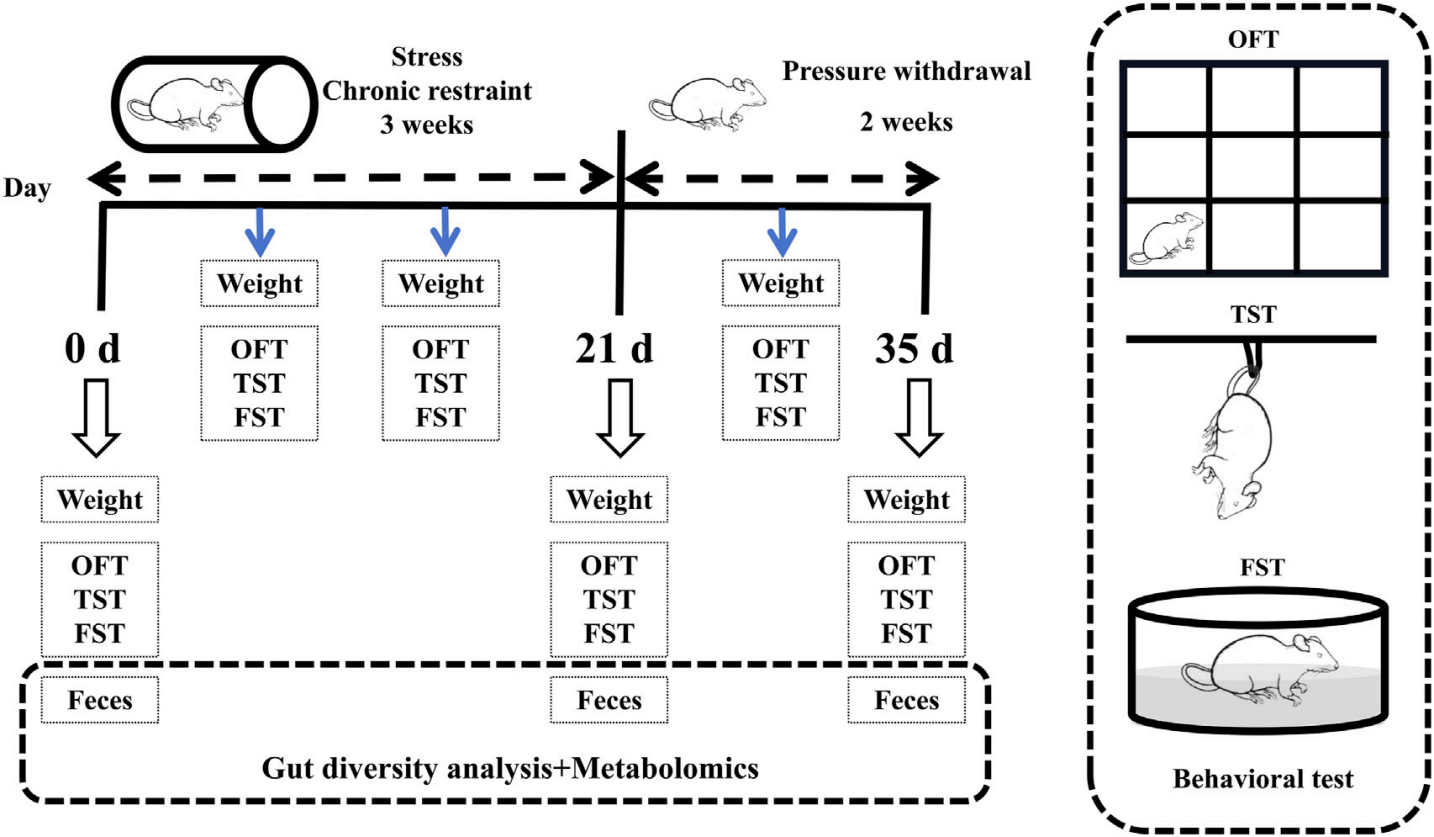
Flow chart of the experiment.

After an adjustment period (a week), 60 mice were randomly divided into 6 groups (n = 10): male control group, female control group, male stress group, female stress group, male stress recovery group, and female stress recovery group. Except for two control groups (Male and Female), mice were exposed to the chronic confining stressor of being bound to a 50 mL centrifuge tube for 6 h (8:30–14:30) per d for 3 weeks [12, 13]. Specifically, the weight of the mice in each group was measured weekly (Figure 1).

### Behavioral tests

The behavioral tests consisted of the open field test (OFT), the tail suspension test (TST), and the forced swim test (FST), which were performed weekly during stress and recovery periods. The OFT followed the procedure described by Choleris et al. [14]. The TST and FST followed the procedures described by O’Leary (2009) and Porsolt (1977), respectively [15, 16]. Prior to the behavioral tests, test animals are allowed an acclimatization period of 1–2 h in the testing environment. Subsequently, the tests are conducted in ascending order of stimulus intensity (1. OFT; 2. TST; 3. FST). The specifics of the behavioral experiment are detailed in Supplementary Material S1.

### Gut fecal metabolite analysis

Following the completion of behavioral testing, mice were euthanized via cervical dislocation. Fecal samples from both male and female mice were collected at three time points: prior to the experiment (0 days), during the experimental period (21 days), and during the stress recovery phase (14 days). Samples were immediately frozen in liquid nitrogen and stored at −80°C. Metabolomics analysis was conducted using LC-MS on 50 mg aliquots of freeze-dried fecal material [17–19]. See Supplementary Material S1 for details.

### Gut microbial diversity analysis

The remaining samples in each group after the metabolomics analysis of the rectal contents were placed in a −80°C refrigerator for DNA extraction. The extracted DNA was used for the 16S rRNA gene analysis of the bacterial population [16, 20]. See Supplementary Material S1 for details.

### Statistical analyses

All data are expressed as the mean ± standard deviation (SD) of 10 or 6 replicates, and ANOVA was performed using a fully randomized trial design. SPSS 22.0 software was used for one-way ANOVA to determine differences between the means [without assuming consistent standard deviation (SD)] and Spearman correlation analysis. P < 0.05 was considered statistically significant. Graphs were drawn using Origin 2021 software.

## Results

### Stress-induced body weight loss is greater in female mice

**FIGURE 2:**
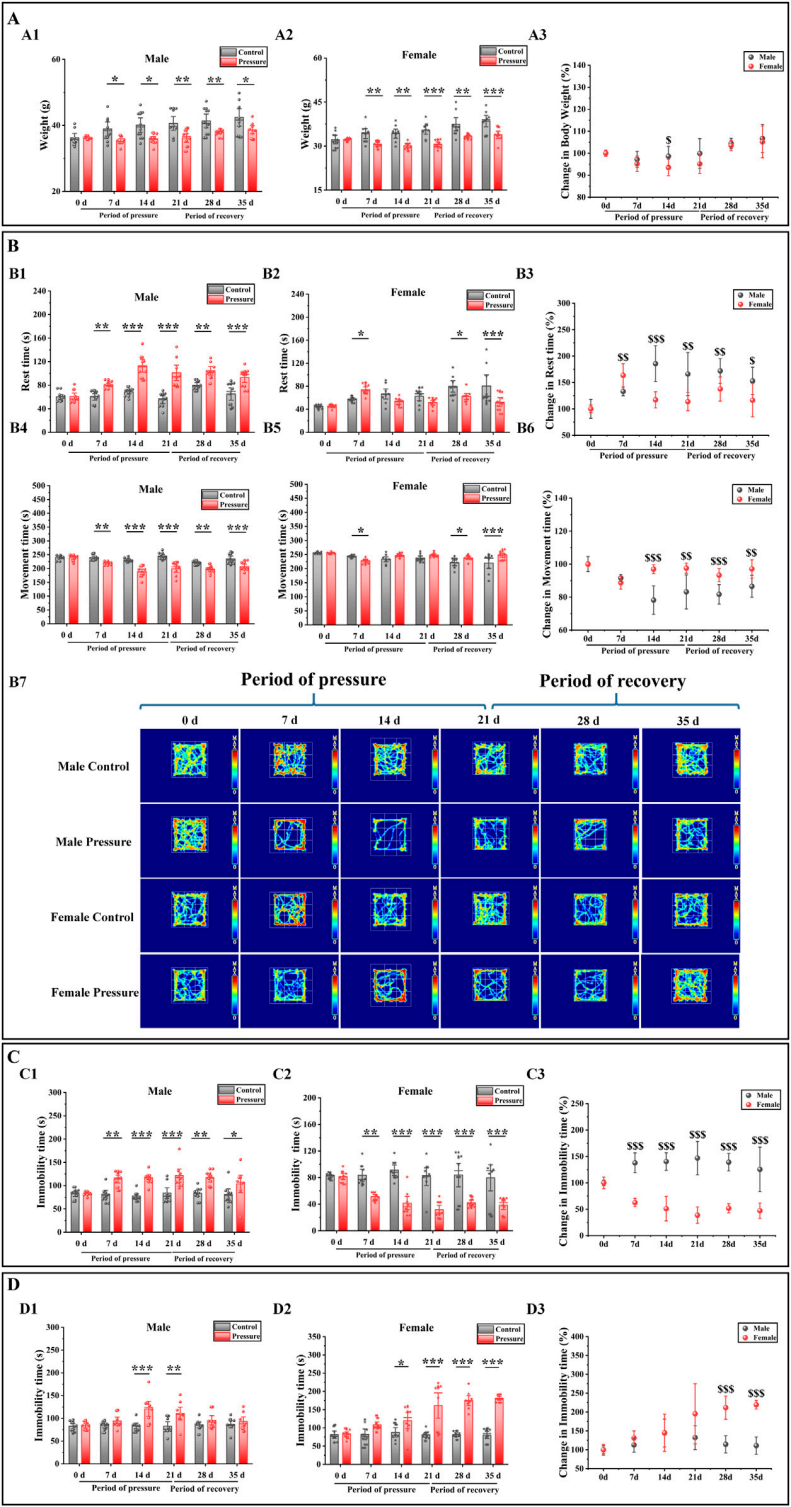
Effects of pressure simulation on mice in **(A)** Weight, **(B)** Rest time and Movement time in OFT, **(C)** TST-IT and **(D)** FST-IT. Note: **(A1, A2)** represent the initial weights of male and female mice, respectively, while **(A3)** denotes the changes in their weights. **(B1, B2)** indicate the RT of male and female mice, respectively, and **(B3)** represents the changes in RT. **(B4, B5)** refer to the MT of male and female mice, with **(B6)** indicating the changes in MT. **(B7)** Illustrates the movement tracks of the OFT for the mice. **(C1, C2)** denote the IT of male and female mice in the TST, with **(C3)** representing the changes in TST-IT. **(D1, D2)** indicate the IT of male and female mice in the FST, and **(D3)** shows the changes in FST-IT. Data are presented as the mean ± SD (n = 10). ^*^p < 0.05, ^**^p < 0.01, and ^***^p < 0.001 indicate significant differences between the control group and the stress group. ^$$^p < 0.01 and ^$$$^p < 0.001 indicate significant differences between the male stress group and the female stress group.

To evaluate the weight alterations in mice elicited by stress stimuli, we conducted a study measuring the body weights of both male and female mice prior to and following exposure to stress. The weight of the mice was measured as shown in Figure 2A, and it was found that in the stress period (7 days, 14 days, and 21 days) and recovery period (28 days and 35 days), the body weight of the male control group mice was significantly lower than that of the male stress group mice (Figure 2A1: 7 days, P < 0.05; 14 days, P < 0.01; 21 days, P < 0.01; 28 days, P < 0.01; 35 days, P < 0.05). The weight change in female mice was similar to that in male mice, and compared with the female control group (Figure 2A2), the weight loss of female mice in the stress group was more significant (7 days, P < 0.01; 14 days, P < 0.01; 21 days, P < 0.001; 28 days, P < 0.01; 35 days, P < 0.05). The weight changes in male and female mice are illustrated in Figure 2A3. Compared to the male stress group mice, the female stress group mice exhibited lower weight changes before stress exposure, during the stress period (7 days, 14 days, and 21 days), and during the recovery period (28 days and 35 days). Notably, a significant difference was observed between male and female mice at 14 days (P < 0.05). Therefore, the observed weight changes prompted us to investigate the corresponding behavioral modifications in the mice.

### Stress exerted a more pronounced influence on the behavior of female mice

To evaluate the impact of stress on mouse behavior, we employed the OFT, TST, and FST to detect behavioral changes in both male and female mice before and after exposure to stress. The OFT results showed that the rest time (RT) of male control group mice was significantly longer than that of male stress group mice during the stress period and recovery period (Figure 2B1: 7 days, P < 0.01; 14 days, P < 0.001; 21 days, P < 0.001; 28 days, P < 0.01; 35 days, P < 0.001), and the movement time (MT) was significantly decreased (Figure 2B4: 7 days, P < 0.01; 14 days, P < 0.001; 21 days, P < 0.001; 28 days, P < 0.01; 35 days, P < 0.001). Compared with the female stress group, the RT of the female control group was significantly increased (Figure 2B2: 7 days, P < 0.05; 28 days, P < 0.05; 35 days, P < 0.001), and the MT was significantly decreased (Figure 2B5: 7 days, P < 0.05; 28 days, P < 0.05; 35 days, P < 0.001). The female stress group was compared with the male stress group (Figure 2B3, B6); the RT change of male mice was significantly higher than that of female mice (14 days, P < 0.001; 21 days, P < 0.01; 28 days, P < 0.01; 35 days, P < 0.05), and the MT was significantly lower than that of female mice (14 days, P < 0.001; 21 days, P < 0.01; 28 days, P < 0.001; 35 days, P < 0.01). However, at 7 days, the RT changes in male mice were significantly lower compared to those in female mice (P < 0.01), while the MT changes were significantly higher. Figure 2B7 shows that the movement trajectory of male control mice increased with time, with little change. In the stress period, the range of motion of the mice tended to be around the periphery, and in the recovery period, the range of motion of the mice began to gradually shift to the center. The motion trajectory of female control mice was similar to that of male control mice. During the stress period, the activity of female stress mice tended to be around the periphery, but the activity of female stress mice was significantly stronger than that of male stress mice. During the recovery period, the activity of female stress mice began to gradually shift to the center, similar to that of male stress mice.

The TST and FST results showed that during the stress period and recovery period, compared with the male control group mice (Figures 2C1, D1), the IT (immobility time) of the male stress group mice in the TST and FST was significantly increased (7 days: P < 0.01; 14 days: P < 0.001, P < 0.001; 21 days: P < 0.001, P < 0.01; 28 days, P < 0.01; 35 days, P < 0.05). Compared with the female control group (Figures 2C2, D2), the TST-IT of female stress group mice was significantly decreased (7 days: P < 0.01; 14 days: P < 0.001; 21 days: P < 0.001; 28 days: P < 0.001; 35 days: P < 0.001), and the FST-IT increased significantly (14 days: P < 0.05; 21 days: P < 0.001; 28 days: P < 0.001; 35 days: P < 0.001). The female stress group was compared with the male stress group (Figures 2C3, D3); the TST-IT changes of male mice was significantly higher than that of female mice (7 days, P < 0.001; 14 days, P < 0.001; 21 days, P < 0.001; 28 days, P < 0.001; 35 days, P < 0.001), and the FST-IT changes was significantly lower than that of female mice (28 days, P < 0.001; 35 ays, P < 0.001). Therefore, based on the outcomes of the behavioral tests conducted on mice, we subsequently investigated the alterations in intestinal microbiota associated with these behaviors.

### Effects of stress on the gut microbiota diversity and function prediction of mice

**FIGURE 3:**
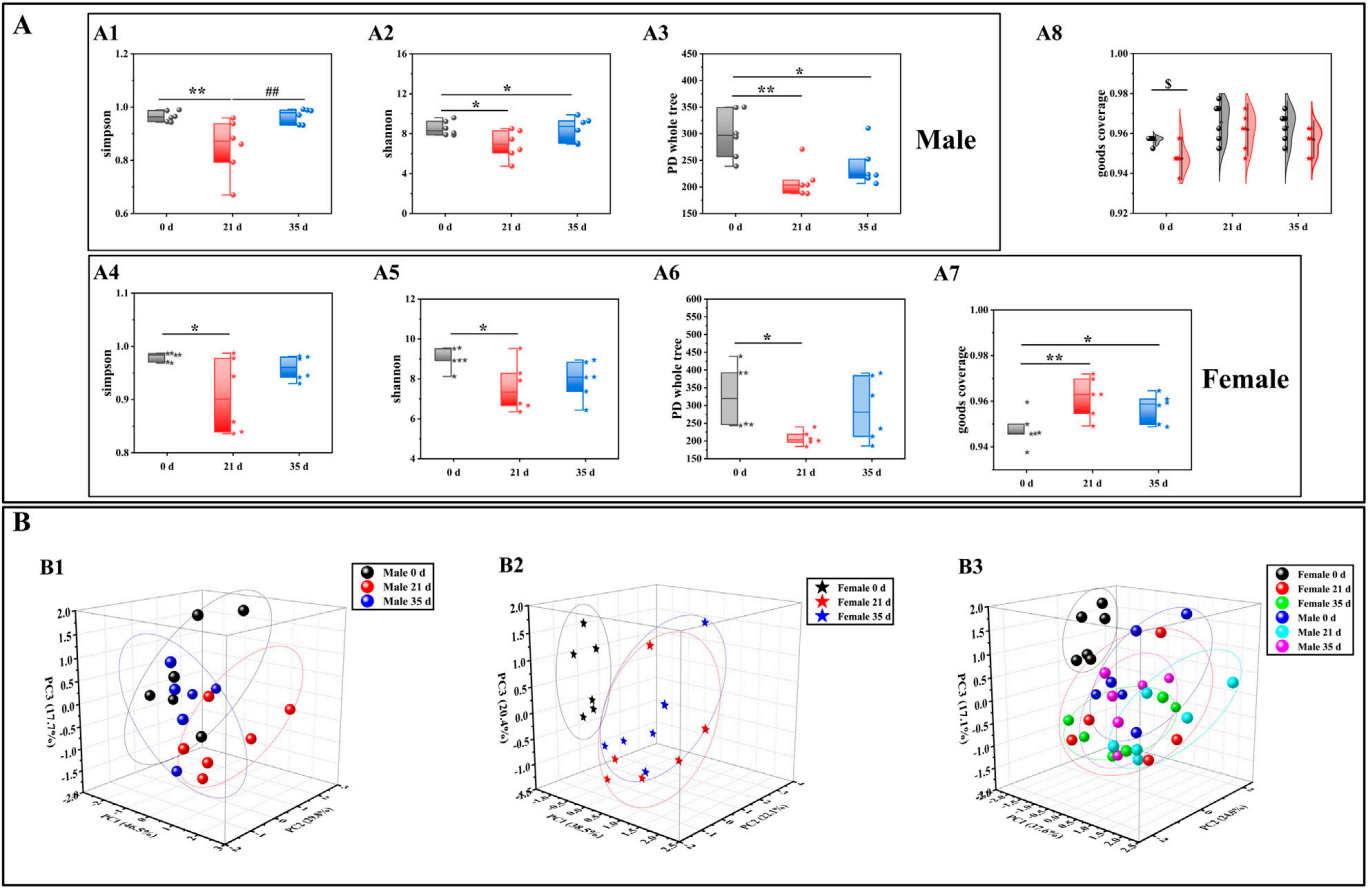
Effects of pressure simulation on the gut microbiota diversity of female and male mice. **(A)**: Alpha diversity analysis; **(B)**: Beta diversity analysis; **(C)**: Cladogram; **(D)**: LDA score; **(E)**: PICRUSt2 one-level pathway annotation results. Note: **(A1–A3)** denote the alpha diversity indices for male mice, while **(A4–A7)** indicate the alpha diversity indices for female mice. **(A8)** represents the combined alpha diversity index for both male and female mice, ensuring comprehensive coverage. **(B1)** refers to the beta diversity analysis for male mice, **(B2)** for female mice, and **(B3)** encompasses the beta diversity analysis for both sexes. Data are expressed as mean ± standard deviation (n = 6). *p < 0.05, **p < 0.01 indicates a significant difference compared with 0days $p < 0.05 indicates a difference between male and female mice.

**FIGURE 4:**
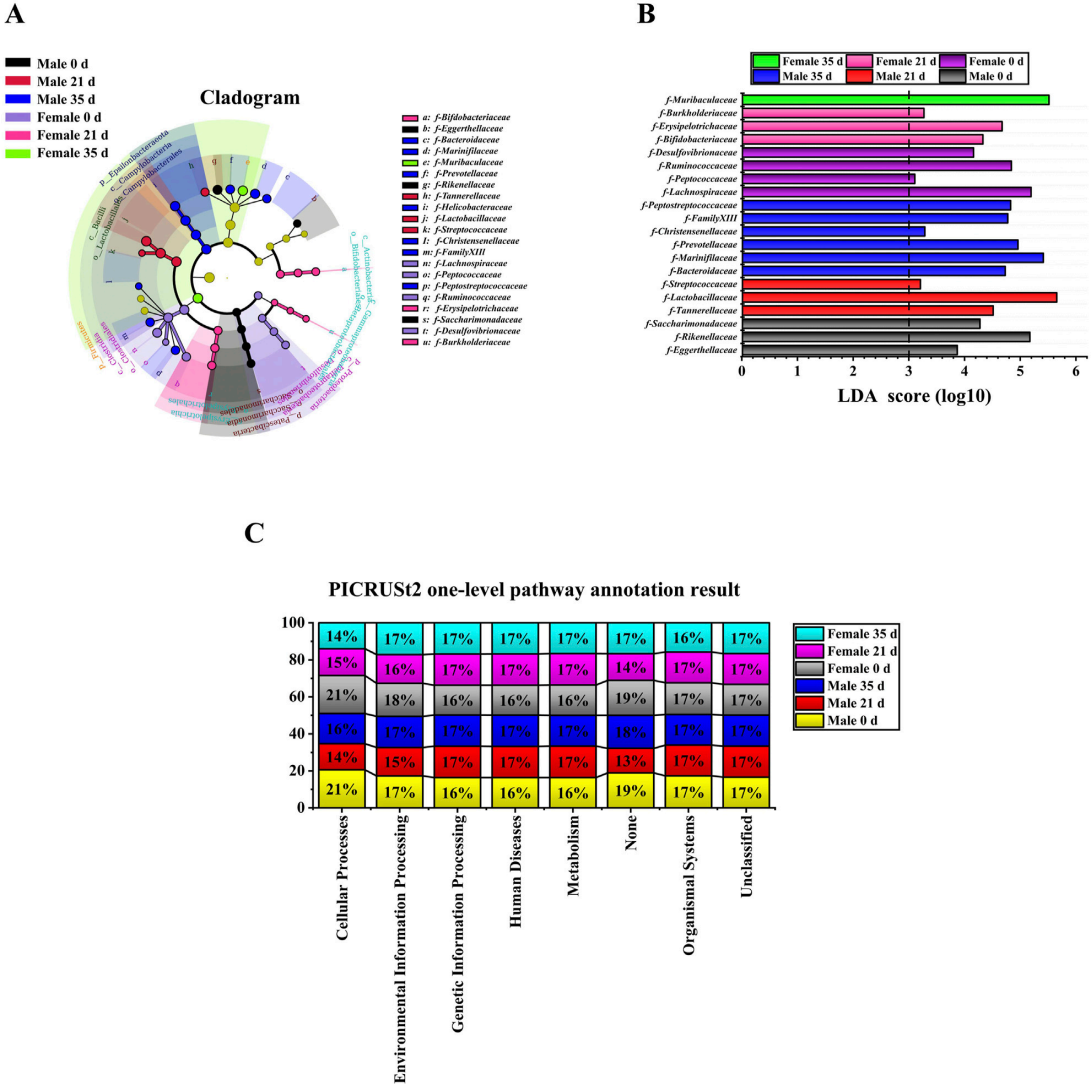
LEfSe analysis and functional prediction of gut microbiota in male and female Mice. **(A)**: Cladogram; **(B)**: LDA score (LDA score≥3); **(C)**: PICRUSt2 one-level pathway annotation results.

To evaluate the alterations in the gut microbiota of mice induced by stress, we initially examined the diversity of the gut microbiota in both male and female mice prior to and following the stress exposure. In the alpha diversity analysis (Figure 3A), including Simpson (male: 21 days vs. 0 days, P < 0.01; 35 days vs. 21 days, P < 0.01. Female: 21 days vs. 0 days, P < 0.05), Shannon (male: 21 days vs. 0 days, P < 0.05; 35 days vs. 0 days, P < 0.05. Female: 21 days vs. 0 days, P < 0.05), PD whole tree (male: 21 days vs. 0 days, P < 0.01; 35 days vs. 0 days, P < 0.05. Female: 21 days vs. 0 days, P < 0.05) and goods coverage (female: 21 days vs. 0 days, P < 0.01; 35 days vs. 0 days, P < 0.05. Male 0 days vs. female 0 days: P < 0.05), the differences between the index groups were statistically significant. Through β diversity analysis (Figure 3B) of the values calculated by Weighted UniFrac, it was found that the samples of each group had obvious differences. Subsequently, we employed LEfSe (Linear discriminant analysis Effect Size) analysis to identify significant differences in the gut microbiota of male and female mice before and after exposure to stress. The cladogram in Figure 4A shows that the study group played an important role in the microbiota. According to the statistical analysis at the species level, *f-Eggerthellaceae*, *f-Rikenellaceae* and *f-Saccharimonadaceae* were the main gut microbiota in the male 0 days group. *f-Tannerellaceae*, *f-Lactobacillaceae* and *f-Streptococcaceae* were the main gut microbiota in the male 21 days group. *f-Bacteroidaceae*, *f-Marinifilaceae*, *f-Prevotellaceae*, *f-Helicobacteraceae*, *f-Christensenellaceae*, *f-Family XIIl* and *f-Peptostreptococcaceae* were the main gut microbiota in the male 35 days group. *f-Lachnospiraceae*, *f-Peptococcaceae*, *f-Ruminococcaceae*, and *f-Desulfovibrionaceae* were the main gut microbiota in the female 0 days group. *f-Bifdobacteriaceae*, *f-Erysipelotrichaceae* and *f-Burkholderiaceae* were the main gut microbiota in the female 21 days group. *f-Muribaculaceae* was the main gut microbiota in the female 35 days group. The histogram of the LDA (linear discriminant analysis) value distribution (Figure 4B) shows the species with LDA scores greater than the set value of 3 and P < 0.05. There were 3 species in males at 0 days, 3 species in males at 21 days and 6 species in males at 35 days. There were 4 species in females at 0 days, 3 species in females at 21 days, and 1 species in females at 35 days. Ultimately, we assessed the biological functional pathways associated with the differential microbiota observed in male and female mice. As shown in Figure 4C, the primary pathways that annotate the gut microbiota were mainly distributed in cellular processes, environmental information processing, genetic information processing, human diseases, metabolism, none, organismal systems, and unclassified. Based on these findings, we subsequently evaluated the alterations in the differential microbiota between male and female mice.

### Stress had a more pronounced impact on the gut microbiota in female mice

**FIGURE 5:**
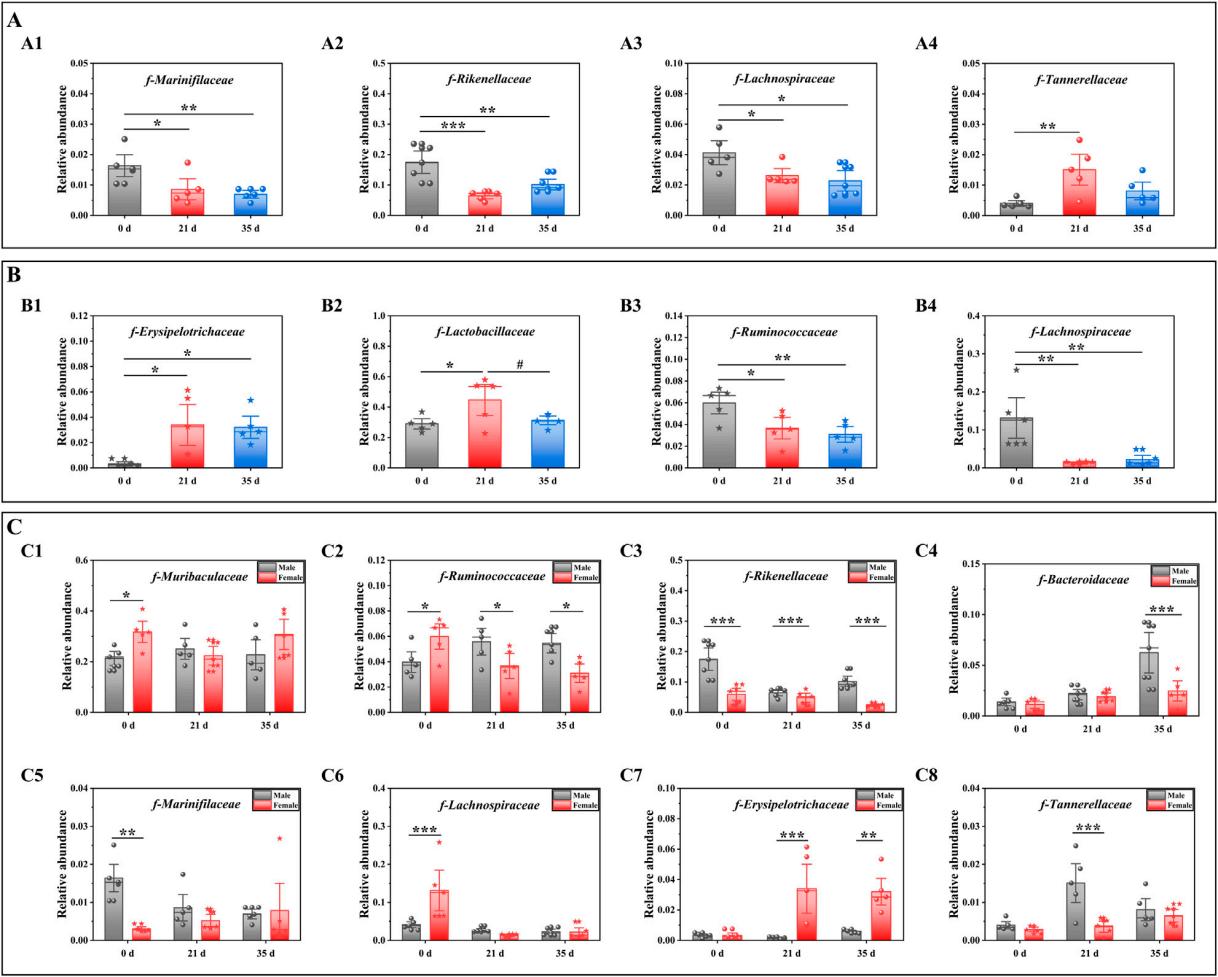
Effects of pressure on gut microbiota at the family level in mice. Note: **(A)** The gut microbiota composition of male mice before and after exposure to stress; A1–A4 denotes varying levels of microbial abundance. **(B)** Family-level comparison of the gut microbiota in female mice before and after exposure to stress; B1–B4 denotes different levels of microbiota abundance. **(C)** Comparative analysis of the gut microbiota between male and female mice before and after exposure to stress; C1–C8 denotes distinct levels of microbiota abundance. Data are expressed as mean ± standard deviation (n = 6). *p < 0.05, **p < 0.01 indicates a significant difference compared with 0 d. #p < 0.05 indicates a significant difference compared with 21 d. $p < 0.05 $$p < 0.01 and $$$p < 0.001 indicates a difference between male and female. Mice.

To evaluate the impact of stress on the gut microbiota of male and female mice, we conducted a comparative analysis of the changes in gut microbiota both between and within the two groups, before and after exposure to stress. Figures 5A–C illustrates the variations in gut microbiota abundance between male and female mice before and after stress exposure at the family level, as determined by one-way ANOVA. For male mice (Figure 5A), intra-group analysis revealed a significant decrease in the relative abundance of *f-Rikenellaceae* (P < 0.05), *f-Lachnospiraceae* (P < 0.001), and *f-Marinifilaceae* (P < 0.05) at 21 days compared to 0 days. Conversely, the relative abundance of *f-Tannerellaceae* significantly increased (P < 0.01). Between 21 days and 35 days, the relative abundance of *f-Lachnospiraceae*, *f-Marinifilaceae*, and *f-Tannerellaceae* decreased, while that of *f-Lachnospiraceae* increased without reaching statistical significance. Over the entire period from 0 days to day 35 days, there was a significant reduction in the relative abundance of *f-Rikenellaceae* (P < 0.01), *f-Lachnospiraceae* (P < 0.01), and *f-Marinifilaceae* (P < 0.05), whereas the increase in *f-Tannerellaceae* did not reach statistical significance. For female mice (Figure 5B), intra-group analysis showed a significant increase in the relative abundance of *f-Erysipelotrichaceae* (P < 0.05) and *f-Lactobacillaceae* (P < 0.01) at 21 days compared to 0 days, while the relative abundance of f-Ruminococcaceae (P < 0.01) and f-Lachnospiraceae (P < 0.01) significantly decreased. From 21 days to 35 days, the relative abundance of *f-Erysipelotrichaceae*, *f-Lactobacillaceae*, and *f-Ruminococcaceae* decreased, although only *f-Lachnospiraceae* showed a significant increase (P < 0.01). Over the entire period from 0 days to 35 days, the relative abundance of *f-Erysipelotrichaceae* (P < 0.05) and *f-Lactobacillaceae* increased, while that of *f-Ruminococcaceae* (P < 0.01) and *f-Lachnospiraceae* (P < 0.01) significantly decreased.

Figure 5C shows the differences in the gut microbiota of mice before and after stress. At 0 days, the relative abundances of *f-Muribaculaceae* (P < 0.05), *f-Ruminococcaceae* (P < 0.05) and *f-Lachnospiraceae* (P < 0.001) decreased. The relative abundances of *f-Rikenellaceae* (P < 0.001), *f-Bacteroidaceae*, *f-Erysipelotrichaceae*, *f-Tannerellaceae* and *f-Marinifilaceae* (P < 0.01) increased. At 21 days, the relative abundances of *f-Muribaculaceae*, *f-Ruminococcaceae* (P < 0.05), *f-Rikenellaceae* (P < 0.01), *f-Bacteroidaceae*, *f-Tannerellaceae* (P < 0.001) and *f-Marinifilaceae* increased, while the relative abundance of *f-Erysipelotrichaceae* (P < 0.001) decreased. At 35 days, the relative abundances of *f-Ruminococcaceae* (P < 0.05), *f-Rikenellaceae* (P < 0.001), and *f-Tannerellaceae* increased, and the relative abundances of *f-Muribaculaceae*, *f-Marinifilaceae* and *f-Erysipelotrichaceae* decreased (P < 0.01). Based on the outcomes from the diverse flora, we subsequently conducted an analysis of the alterations in gut metabolites.

### Effects of stress on gut metabolism in mice

**FIGURE 6:**
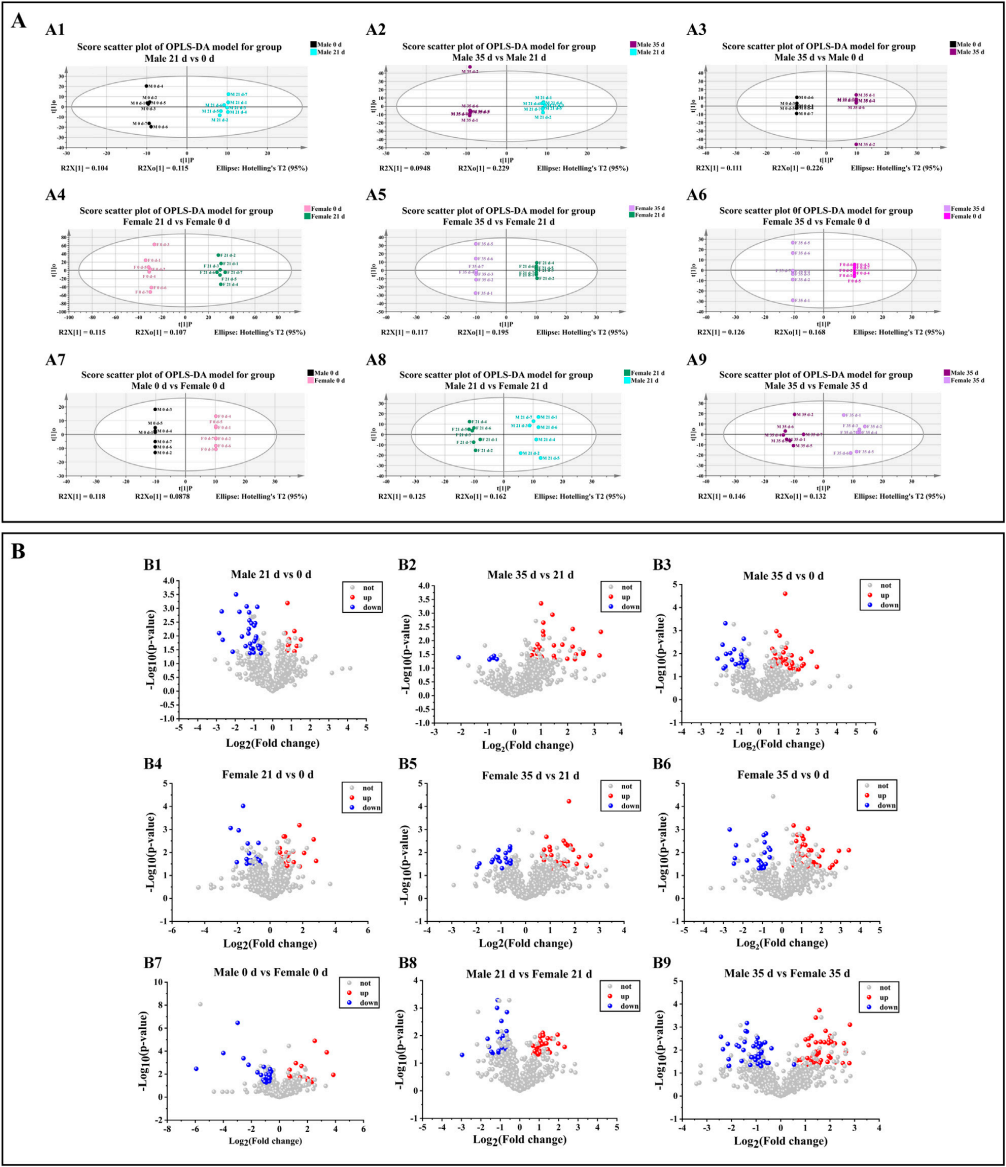
Effects of pressure on gut metabolism in mice. **(A)** Scatter plot of the OPLS-DA model. A1–A3 denotes the scatter plot of OPLS-DA model of metabolite comparison before and after stress in male mice. A4–A6 denotes the scatterplot of OPLS-DA model of metabolite comparison before and after stress in female mice. A7–A9 denotes a scatter plot of the OPLS-DA model in which metabolites in female and male mice are compared before and after stress. **(B)** Volcano map. B1–B3 denotes the volcanic map of metabolites before and after stress in male mice, B4–B6 denotes the volcanic map of metabolite comparison before and after stress in female mice, B7–B9 denotes the volcanic map of metabolites in female and male mice before and after stress (n = 6).

To evaluate the impact of stress on intestinal metabolites in male and female mice, we performed a comparative analysis of the alterations in intestinal metabolites within each sex group, both before and after exposure to stress. The scatter plot of the OPLS-DA (orthogonal partial least squares discriminant analysis) model of mice in each group is shown in Figure 6A. The sample differentiation was very significant, and all samples were within the 95% confidence interval. The screening conditions for DMs (differential metabolites) were VIP >1 and P value <0.05. In addition, the results of screening upregulated DMs in each group of mice were visualized by a volcanic map (FC > 1.5; FC < 0.67, indicating downregulation). In male mice (Figures 6B1–3), compared to 0 days, 10 DMs were up-regulated and 30 DMs were down-regulated by 21 days. Between 21 days and 35 days, 29 DMs were up-regulated while 8 DMs were down-regulated. Over the entire period from 0 days to 35 days, 38 DMs were up-regulated and 18 DMs were down-regulated. In female mice (Figures 6B4–6), relative to 0 days, 21 DMs were up-regulated and 22 DMs were down-regulated by 21 days. From 21 days to 35 days, 32 DMs were up-regulated and 19 DMs were down-regulated. Overall, from 0 days to 35 days, 39 DMs were up-regulated and 22 DMs were down-regulated. When comparing male and female mice (Figures 5B7–9), at 0 days, 21 DMs were up-regulated and 28 DMs were down-regulated in males compared to females. By 21 days, 26 DMs metabolites were up-regulated and 26 DMs were down-regulated in males relative to females. At 35 days, this difference increased to 50 DMs up-regulated and 41 DMs down-regulated in males compared to females. Based on the findings from various gut metabolites, we subsequently conducted an in-depth analysis of the associated gut metabolic pathways.

### Effects of stress on metabolic pathways and metabolites in mice

**FIGURE 7:**
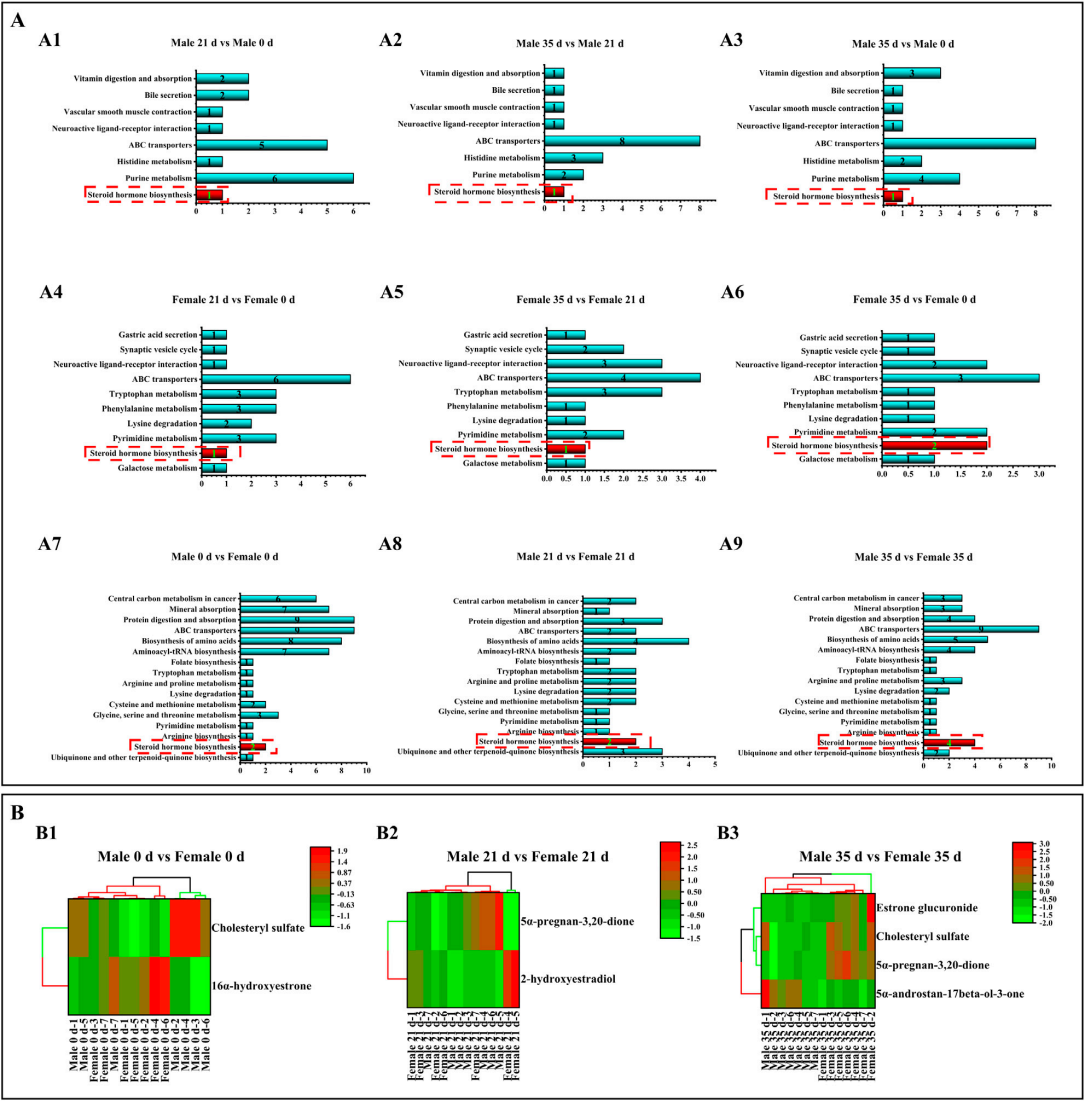
Effects of pressure on metabolic pathways and metabolites in mice. Note: **(A)** Metabolic pathways. A1–A3 denotes the metabolic pathways of male mice compared with DMs before and after stress. A4–A6 denotes the metabolic pathway of DMs compared before and after stress in male mice. A7–A9 denotes the metabolic pathway of DMs compared before and after stress in male and female mice. **(B)** DMs cluster analysis. B1 denotes DMs cluster analysis of male and female mice at 0 days; B1 denotes DMs cluster analysis of male and female mice at 21 days. B1 denotes DMs cluster analysis of male and female mice for 35 days (n = 6).

To evaluate the metabolic pathways involved in the stress response, the KEGG (Kyoto Encyclopedia of Genes and Genomes) was analyzed in male and female mice both before and after exposure to stress. The results of differential metabolite pathways were analyzed in a bar chart (Figure 7A). The DMs enrichment of male and female mice at three time points involved the same 16 pathways. DMs enrichment in both male and female mice at three time points (0 days, 21 days, and 35 days) involved the same 16 pathways, with a particular emphasis on the steroid hormone biosynthesis pathway. Due to the limited number of DMs (only 1-2) identified in the comparison between male and female mice, clustering analysis was not feasible. Consequently, we conducted inter-group comparisons between males and females and performed DM cluster analysis at the three specified time points (Figure 7B). Male mice were compared to female mice. On 0 days, 1 DM was up-regulated and 1 DM was down-regulated. By 21 days, 1 DM remained up-regulated while another DM was down-regulated. On 35 days, 1 DM was up-regulated and 3 DMs were down-regulated.

### Correlation analysis of differential gut microbiota and DMs

**FIGURE 8:**
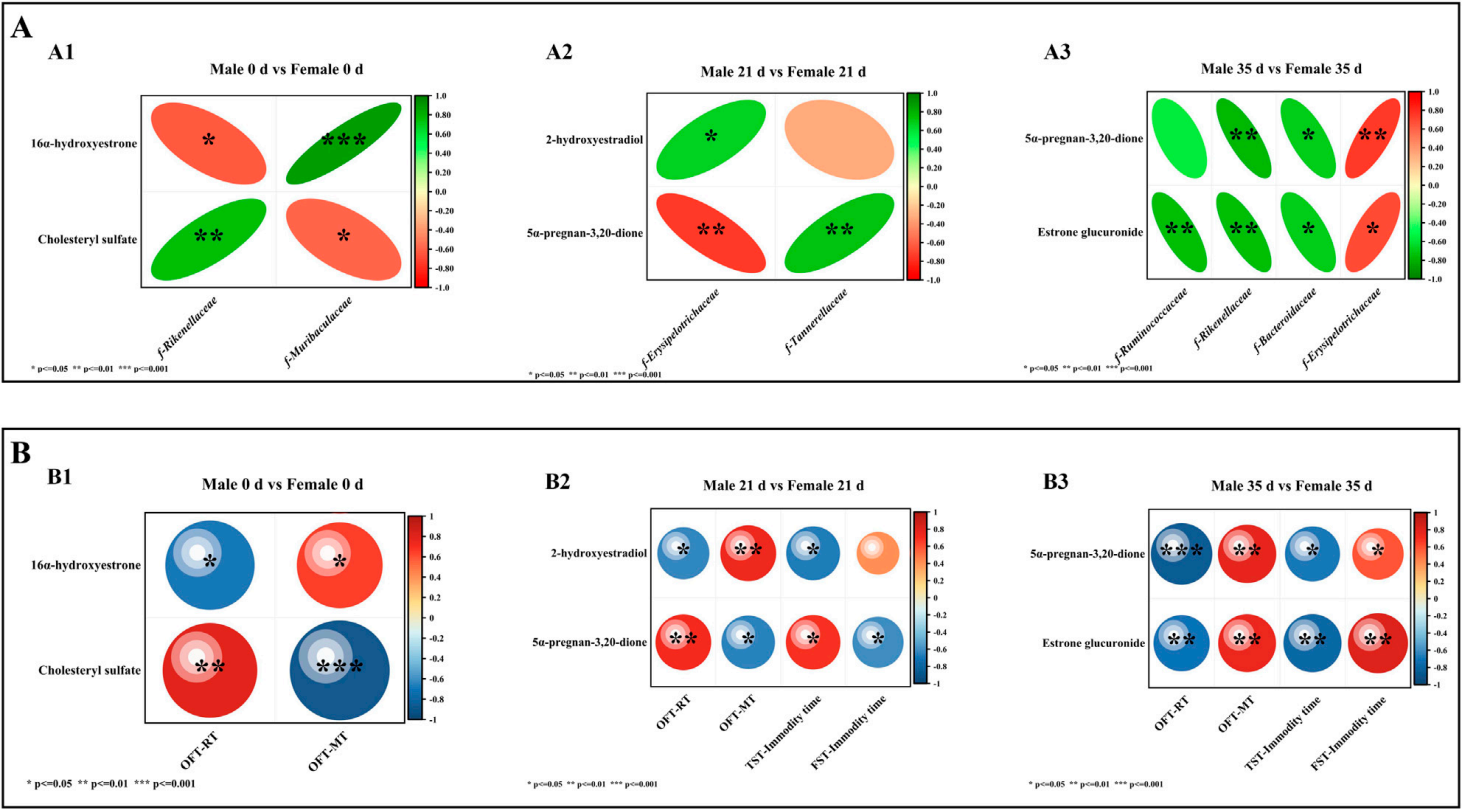
Spearman correlation analysis. Note **(A)** Correlation analysis between differential microbiota and DMs. A1 denotes the correlation analysis between 0 days of differential microbiota and DMs; A2 denotes the correlation analysis between 21 days of different microbiota and DMs; A3 denotes the correlation analysis between 35 days of different microbiota and DMs. **(B)** Correlation analysis between DMs and behavior. B1 denotes the correlation analysis between DMs and behavior at 0 days; B2 denotes the correlation analysis between DMs and behavior in 21 days; B3 denotes the correlation analysis between DMs and behavior in 35 days (n = 6).

To evaluate the association between metabolites and gut microbiota, Spearman correlation analysis was conducted to examine the relationship between the gut microbiota and DMs in male and female mice both before and after stress exposure. The results were subsequently compared between the sexes, as illustrated in Figure 8A. At 0 days, 16α-hydroxyestrone was negatively correlated with *f-Rikenellaceae* (P < 0.05) and positively correlated with *f-Muribaculaceae* (P < 0.001); cholesteryl sulfate was positively correlated with *f-Rikenellaceae* (P < 0.01) and negatively correlated with *f-Muribaculaceae* (P < 0.05). At 21 days, 2-hydroxyestradiol was positively correlated with *f-Erysipelotrichaceae* (P < 0.05); 5α-pregnane-3,20-dione was positively correlated with *f-Tannerellaceae* (P < 0.01) and negatively correlated with *f-Erysipelotrichaceae* (P < 0.01). At 35 days, 5α-pregnane-3,20-dione was positively correlated with *f-Erysipelotrichaceae* (P < 0.01) and negatively correlated with *f-Rikenellaceae* (P < 0.01) and *f-Bacteroidaceae* (P < 0.05); estrone glucuronide was negatively correlated with *f-Ruminococcaceae* (P < 0.01), *f-Rikenellaceae* (P < 0.01), and *f-Bacteroidaceae* (P < 0.05) and positively correlated with *f-Erysipelotrichaceae* (P < 0.05).

### Correlation analysis of DMs and behavior

To evaluate the relationship between metabolites and behavior, Spearman correlation analysis was conducted on the behavioral data and DMs of both male and female mice before and after exposure to stress. The results were subsequently compared between the two sexes, as illustrated in Figure 8B. At 0 days, 16α-hydroxyestrone was negatively correlated with OFT-RT (P < 0.05) and positively correlated with OFT-MT (P < 0.05), and cholesteryl sulfate was positively correlated with OFT-RT (P < 0.01) and negatively correlated with OFT-MT (P < 0.001). At 21 days, 2-hydroxyestradiol was negatively correlated with OFT-RT (P < 0.05) and TST-IT (P < 0.05) and positively correlated with OFT-MT (P < 0.01); 5α-pregnane-3,20-dione was positively correlated with OFT-RT (P < 0.01) and TST-IT (P < 0.05) and negatively correlated with OFT-MT (P < 0.05) and FST-IT (P < 0.05). At 35 days, 5α-pregnane-3,20-dione and estrone glucuronide were positively correlated with OFT-RT (P < 0.001; P < 0.01), and TST-IT (P < 0.05; P < 0.01) and negatively correlated with OFT-MT (P < 0.01; P < 0.01) and FST-IT (P < 0.05; P < 0.01).

## Discussion

Given the clinical phenomenon of sex differences in depression [21], this study explored the reasons for the differences in stress tolerance and recovery ability between male and female mice. We adopted the CRS model [22] and exposed mice to 21 days of continuous stress for 6 h to simulate a stressful environment. Clinically, weight loss is observed in depressed patients, and the degree of weight loss in women may be greater [23]. Our experimental results also showed this phenomenon. After 21 days of stress simulation, the weight change in female mice was similar to that in male mice, but the weight loss of female mice was more obvious. After 14 days of stress cessation, female mice also had lower weight recovery ability than male mice. The behavioral test results showed that male mice exhibited lower activity and increased resting time during stress compared to female mice. Interestingly, however, the female mice showed higher exercise time on the TST and lower IT on the FST. We speculate that female mice and male mice exhibit different depressive phenotypes [24, 25] due to differences in sex personality and physiological metabolism [9, 26, 27] during stress and recovery, which may be the reason why female mice exhibited anxiety-like behaviors in the OFT and TST. Male mice began to return to desperate behavior, but female mice exhibited less recovery and increased depressive behavior. This is also similar to the clinical phenomenon that the recurrence rate of MDD in women is four times that of men [24].

The differences in depressive behavior and phenotype between female and male mice may be related to the regulation of sex hormones [9, 28]. Estradiol is a sex hormone that is crucial for regulating emotions. *Bacteroides thetaiotaomicron* and *Clostridia* can degrade estradiol through the metabolism of 3β-steroid dehydrogenase, which can induce depression in women [9]. Currently, behavioral differences caused by stress are known to be significantly related to the gut microbiota and its metabolites [29, 30]. At the same time, there are also significant sex differences in the composition of the microbiota. According to existing studies, men and women show differences in susceptibility, diagnosis, treatment and recovery of most diseases related to the microbiota [31]. The occurrence and development of depression cannot be separated from immunity and metabolism, and the microbiota plays an important role in immune metabolism [32]. However, we do not yet know what causes the behavioral differences between male and female mice during stress and recovery, or how the gut is involved in this process. Therefore, we performed microbiota 16S sequencing and metabolomics analysis of the gut contents.

Our results demonstrated that the relative abundance of *f-Ruminococcaceae* and *f-Lachnospiraceae* significantly decreased in both male and female mice during the 21 days stress period. This reduction may represent a common response of the murine gut microbiota to stress [33]. However, sex-specific differences in gut microbiota have been documented [34]. In our study, we observed distinct variations in the microbial composition between male and female mice. Prior to stress exposure (at 0 days), the relative abundance of *f-Rikenellaceae* was higher in male mice compared to females, whereas the relative abundance of f-Ruminococcaceae was lower in males than in females. Following 21 days of stress stimulation, the relative abundance of *f-Rikenellaceae* decreased, with a more pronounced reduction observed in female mice. Notably, the relative abundance of *f-Ruminococcaceae* exhibited opposite trends by sex, decreasing in females while increasing in males. Two weeks after the cessation of stress, the relative abundance of *f-Rikenellaceae* and *f-Ruminococcaceae* began to recover in male mice, whereas it continued to decline in females. In 2015, a study from Ohio State University found that in boys, extroverted personality traits were associated with the abundance of *Rikenellaceae* and *Ruminococcaceae*. *Ruminococcaceae* is one of the main bacterial groups that produces butyric acid, an anti-inflammatory short-chain fatty acid that nourishes the gut mucosa. In girls, the abundance of *Rikenellaceae* seems to influence fear propensity [35]. This study is consistent with our findings of behavioral differences between females and males before stress, during stress, and during recovery. We speculate that the differences in the abundance of *Rikenellaceae* and *Ruminococcaceae* may be the reason for the different behaviors of female and male mice. Moreover, we found that the relative abundance of *f-Erysipelotrichaceae* in female mice began to increase after 21 days of stress and remained high after 14 days of recovery. Studies have found that *Erysipelotrichaceae* may be a driving factor in exercise performance [36], which is consistent with our behavioral results that female mice exhibit strong anxious exercise behaviors. Interestingly, several studies have demonstrated that supplementation with butyrate or butyrate-producing gut flora alleviates depressive symptoms in mice. Ari et al. [37] reported that long-term administration of β-hydroxybutyric acid (3HB) significantly reduced anxiety-like behavior in rats. An Italian research team observed in an autism model that maternal supplementation with butyrate during pregnancy and lactation increased butyrate levels in offspring and mitigated neuro developmental abnormalities and autism-like behaviors [38]. Furthermore, supplementation with certain butyrate-producing gut flora has been shown to improve depressive symptoms in women [33, 39, 40]. Consequently, we hypothesize that the disparity in the abundance of *Rikenellaceae* and *Ruminococcaceae* may influence butyrate metabolism, potentially contributing to differences in stress resilience between female and male mice. However, due to the limitations of our study, further research is required to substantiate this hypothesis.

Excitingly, in our gut metabolomics analysis, we found that the DMs between stress and recovery in male and female mice were concentrated in the steroid hormone biosynthesis pathway, which is involved in estrogen synthesis. Estrogen metabolism is closely related to the occurrence of MDD [9, 27]. Increasing research evidence shows that estrogen regulates neurotransmitters in the brain through estrogen receptor (GPER) signals, thereby affecting cognition, behavior, and emotion [41]. 16α-Hydroxyestrone is one of the metabolites of the female core hormone estradiol and a potent antioxidant [42, 43]. The relative content of 16 α-hydroxyestrone in female mice was higher than that in male mice at 0 days before stress. In Spearman association analysis, 16α-hydroxyestrone was positively correlated with *f-Ruminococcaceae* (P < 0.001) and OFT-MT and negatively correlated with OFT-RT. Therefore, we surmised that 16α-hydroxyestrone induced initial high motility in female mice, which may be related to the higher gut antioxidant and anti-inflammatory capacity of females than males.

Progesterone and estrogen jointly maintain the dynamic balance of the brain, and progesterone balances the excitatory effect of estrogen and plays a role in sedation and anti-anxiety [44, 45]. Progesterone has been shown to have anti-inflammatory effects in neuronal injury and infection models [46, 47]. In addition, many metabolites of progesterone have physiological activities and participate in human emotional regulation [48]. 5α-Pregnane-3,20-dione, a progesterone metabolite, induces increased activity in ovariectomized rats [49]. Estradiol-17β is hydroxylated to form 2-hydroxyestradiol-17β, a neuroprotective estrogen [50].

After 21 days of stress simulation, the relative content of 5α-pregnane-3,20-dione in female mice was lower than that in male mice, while 2-hydroxyestradiol-17β increased. Association analysis of DMs with microbiota and behavior showed that *f-Erysipelotrichaceae* was positively correlated with 2-hydroxyestradiol (P < 0.05) but negatively correlated with 5α-pregnane-3,20-dione (P < 0.01). Pregnane-3,20-dione was positively correlated with OFT RT (P < 0.05) and TST-IT (P < 0.05). 2-Hydroxyestradiol was negatively correlated with OFT RT (P < 0.05) and TST-IT (P < 0.05). Therefore, we speculate that an increase in the relative abundance of *f-Erysipelotrichaceae* in female mice drives stronger anxiety-like behavior and a decrease in 5α-pregnane-3,20-dione levels, resulting in increased FST-IT and increased depression-like behavior. The increase in 2-hydroxyestradiol-17β may reflect the resistance of female mice to stress. Interestingly, female mice performed differently in the two experiments that measured desperate behavior, showing greater struggle in the TST and greater desperation in the FST than male mice. We speculate that this may be related to the cognitive and physical differences between male and female mice. Female mice have lower physical capacity than male mice and are lighter in weight, so they are more likely to sway when suspended, while resistance in water consumes energy and induces females to be more likely to stay still. At the same time, females have stronger cognitive ability than males [51]. In our initial experiment, females had stronger exploration ability than males, and it was easier for females to understand despair in water. However, we cannot yet explain the biological mechanism of this phenomenon. After 2 weeks of stress withdrawal, male mice produced lower levels of 5α-pregnane-3,20-dione and estrone glucuronide than female mice. Estrone glucuronide is the downstream metabolite of estrone, which is also one of the indicators used to predict depression in women and is related to sensitivity to negative emotions [52]. In the association analysis, 5α-pregnane-3,20-dione and estrone glucuronide were positively correlated with *f-Erysipelotrichaceae*, OFT-RT (P < 0.001; P < 0.01) and TST-IT (P < 0.05; P < 0.01). The relative levels of 5α-pregnane-3,20-dione and *f-Erysipelotrichaceae* in female mice began to increase, which was consistent with increased anxious behavior in females. In addition, increased levels of estrone glucuronide may induce increased negative emotional sensitivity in female mice. We hypothesized that after stress withdrawal, the sex hormone metabolism of female mice continued to be disturbed, while depression and anxiety increased.

## Conclusion

In mammals, there are significant sex differences in mental disorders such as depression, autism, and Alzheimer’s disease. We used the CRS model to simulate the persistent stress in the environment that human beings have been exposed to for a long time and explored the differences between male and female mice at three time points (0 days before the stress, 21 days during the stress period, and 35 days during the recovery period) in behavior, gut microbiota, and gut metabolites to find biomarkers in different periods. The experimental results showed that before stress, females exhibited strong activity, a weak ability to despair and struggle, anxiety-prone behavior, *f-Rikenellaceae* and *f-Ruminococcaceae* gut microbiota and 16α-hydroxyestrone, which were significantly different from males. After 21 days of stress, female anxiety and depression-like behavior worsened, gut metabolism and microbiota were disrupted, and there were marked differences in the microbiota (*f-Erysipelotrichaceae*) and metabolites (5α-pregnane-3,20-dione and 2-hydroxyestradiol). After 14 days of stress relief, male depression-like behavior began to recover, while females exhibited exacerbated estrogen metabolism (5α-pregnane-3,20-dione and estrone glucuronide) and gut microbiota (*f-Erysipelotrichaceae*) disorders and aggravated depression-like and anxiety-like behaviors. We hope that this study can provide basic evidence for the phenomenon of sex differences in depression and target markers for the treatment of female depression.

## Data Availability

The datasets presented in this study can be found in online repositories. The names of the repository/repositories and accession number(s) can be found in the article/Supplementary Material.
