# Supplementary material for: A study on the differences in the gut microbiota and metabolism between male and female mice in different stress periods
Source: Exp Biol Med (Maywood). 2025 Feb 11;250:10204. doi: 10.3389/ebm.2025.10204 (PMC11851196; doi:10.3389/ebm.2025.10204)
Supplement: Supplementary file 1 [file DataSheet1.pdf]

Test method details

Open field test

After the stress ended, the opening experiment was used to evaluate the voluntary activity and anxiety level of mice [14]. The mice were moved to the opening experiment room 60 min before the experiment to adapt to the environment in advance. During the experiment, the mice were removed from the cage and placed in the center of the opening experiment device(500 × 500 × 415mm) in the behavioral laboratory, and the shade was quickly drawn. In operating software record number, date, status in mice after open the recording system, select scratchable latex mode, central area ratio is 0.5, through the opening at the top of the equipment connected to the camera and monitor records within 5 min of mice activity and calculate the percentage residence time in central region(central time / 300 s), the central level movement percentage(central horizontal distance/horizontal movement distance). After the experiment, the mice were put back into the cage, and 70% ethanol was used to wipe the experimental apparatus thoroughly and dry with paper towels.

Tail suspension test

The tail suspension test (TST) was used to evaluate the helplessness state of mice [15]. After adaptation to the test environment for 1 h, acoustically and visually isolated mice were suspended by their tail with adhesive tape (1–2 cm distance from the tail tip). The suspended mice were positioned 10 cm above the tabletop for 6 min. The immobility(completely motionless) time of mice was recorded during the last 5 min of the total suspended time by a double-blind timer.

Forced swimming test

The forced swimming test (FST) is another common method to evaluate the helplessness state of mice[16]. In this test, mice were individually placed in a cylindrical glass container with a water depth of 10 cm, a diameter of 14 cm, and a water temperature of 23 ± 2°C and then exposed to an FST session for 6 min. The whole process was recorded by a high-definition digital camera. The immobility time of mice, defined as the time when the mouse stops struggling, floats, and swims slightly to keep its head above water, were recorded by a double-blind timer during the final 4 min.

Gut fecal metabolite analysis

Following the completion of behavioral testing, mice were euthanized via cervical dislocation. Fecal samples from both male and female mice were collected at three time points: prior to the experiment (0 d), during the experimental period (21 d), and during the stress recovery phase (14 d). Samples were immediately frozen in liquid nitrogen and stored at −80°C. For LC–MS detection and analysis, 50 mg of the lyophilized sample were accurately weighted, 800 µL of 80% methanol and 5 µL of dichlorophenylalanine (2.8 mg/ml) were added, and the sample was vortexed for 30 s to mix 30 Hz, 120 s. Samples were sonicated at 4 °C for 15 minutes, left to stand at -20 °C for 1 h, and centrifuged at 12000 rpm for 15 min at 4 °C. Then, 200 µL of supernatant was aspirated and transferred into a sample vial. (I) The on-board detection instrument analysis platform was an LC–MS (Thermo, Ultimate 3000LC, Q Exactive) with a C18 chromatographic column (Hyper Gold C18, 100 x 2.1 mm, 1.9 µm). The chromatographic separation conditions were as follows: column temperature, 45 °C; flow rate, 0.35 mL/min; mobile phase composition, A: water + 5% acetonitrile + 0.1% formic acid, B: acetonitrile + 0.1% formic acid; injection volume, 10 µL; and autosampler temperature, 4 °C. (II) The mass spectrometry detection parameters in positive mode were as follows: heater temperature, 300 °C; sheath gas flow rate, 45 arb; auxiliary gas flow rate, 15 arb; tail gas flow rate, 1 arb; electrospray voltage, 3.0 kV; capillary temperature, 350 °C; and S-Lens RF level, 30%. (III) Compound Discoverer software (Thermo Company) was used to extract and preprocess the LC/MS detection data and organize them into a two-dimensional data matrix, including retention time, molecular weight, observation amount (sample name), and peak intensity. (IV) Data preprocessing included univariate statistical analysis with Student's t test, multivariate statistical analysis with orthogonal partial least squares-discriminant analysis (OPLS-DA), KEGG annotation of differential metabolites, metabolic pathway analysis and hierarchical clustering analysis[18-20].

Gut microbial diversity analysis

After the stress simulations, fresh rectal contents of the mice were collected, frozen quickly in liquid nitrogen, and stored in a refrigerator at −80 °C for subsequent DNA extraction. Microbial DNA was extracted using the E.Z.N.A. Stool DNA Kit (Omega Biotek, Norcross, GA, United States) according to the manufacturer's protocols, after which the extracted DNA was stored at −80 °C until further use. The primers 338F

(5′-CTCCTACGGGAGGCAGCAG-3′) and 806R (5′-GGACTACHVGGGTWTCTAAT-3′) were utilized to amplify the V3–V4 region of the bacterial 16S rDNA gene. The PCR amplification procedure was 95 °C for 3 min; 27 cycles of 95 °C for 30 s, 55 °C for 30 s, and 72 °C for 45 s; and a final extension at 72 °C for 10 min. Approximately 420 bp of amplified fragments was obtained per sample. PCRs were performed on 20 μl samples, containing 4 μl of 5 × FastPfu Buffer, 2 μl of 2.5 mM dNTPs, 0.8 μl of 5 μM primers, 0.4 μl of FastPfu Polymerase, and 10 ng of template DNA, via a PCR system (ABI GeneAmp 9700, Applied Biosystems, Inc., MA, United States). Using the MiSeq platform, paired-end data of 2 × 300 bp were obtained by sequencing. Long sequences were obtained by splicing, and 16S analysis was performed.

The original sequence was quality filtered using Trimmomatic software (version 0.39) and spliced by FLASH software with the following criteria: 1) setting a 50 bpm sliding window, 300 bpm reads were truncated at any site over this window with an average quality score < 20, discarding the truncated reads that were shorter than 50 bp; 2) barcodes matching mismatches of exactly 2 nucleotides in primer matching were allowed, and reads containing ambiguous characters were removed; and 3) two-end sequences were spliced according to base overlap, and overlaps longer than 10 bp and unspaced sequences were removed. Reads that could not be assembled were discarded. Operational taxonomic units (OTUs) were clustered with a 97% similarity cutoff using UPARSE (version 7.1 http://drive5.com/uparse/), and chimeric sequences were identified and removed using UCHIME. The taxonomy of each 16S rRNA gene sequence was analyzed by RDP Classifier (http://rdp.cme.msu.edu/) against the Silva (SSU123) 16S rRNA database using a confidence threshold of 70% [16-17].

References

14. Choleris E, Thomas AW, Kavaliers M, Prato FS. A detailed ethological analysis of the mouse open field test: effects of diazepam, chlordiazepoxide and an extremely low frequency pulsed magnetic field. Neurosci Biobehav Rev. 2001;25(3):235-60. doi: 10.1016/s0149-7634(01)00011-2.

15. Cryan JF, Mombereau C, Vassout A. The tail suspension test as a model for assessing antidepressant activity: review of pharmacological and genetic studies in mice. Neurosci

Biobehav Rev. 2005;29(4-5):571-625. doi: 10.1016/j.neubiorev.2005.03.009.

16. Porsolt RD, Le Pichon M, Jalfre M. Depression: a new animal model sensitive to antidepressant treatments. Nature. 1977;266(5604):730-2. doi: 10.1038/266730a0.

17. Amato KR, Yeoman CJ, Kent A, Righini N, Carbonero F, Estrada A, Gaskins HR, Stumpf RM, Yildirim S, Torralba M, Gillis M, Wilson BA, Nelson KE, White BA, Leigh SR. Habitat degradation impacts black howler monkey (Alouatta pigra) gastroGutmicrobiomes. ISME J. 2013;7(7):1344-53. doi: 10.1038/ismej.2013.16.

18. Zhou B, Xiao JF, Tuli L, Ressom HW. LC-MS-based metabolomics. Mol Biosyst. 2012;8(2):470-81. doi: 10.1039/c1mb05350g.

19. Wiklund S, Johansson E, Sjöström L, Mellerowicz EJ, Edlund U, Shockcor JP, Gottfries J, Moritz T, Trygg J. Visualization of GC/TOF-MS-based metabolomics data for identification of biochemically interesting compounds using OPLS class models. Anal Chem. 2008;80(1):115-22. doi: 10.1021/ac0713510.

20. Kanehisa M, Sato Y, Kawashima M, Furumichi M, Tanabe M. KEGG as a reference resource for gene and protein annotation. Nucleic Acids Res. 2016;44(D1):D457-62. doi: 10.1093/nar/gkv1070.
